# Optimization of Flow Rate for Uniform Zinc Phosphate Coating on Steel Cylinders: A Study on Coating Uniformity and Elemental Composition Using Scanning Electron Microscopy (SEM)

**DOI:** 10.3390/ma18112442

**Published:** 2025-05-23

**Authors:** Yu-Nah Jeong, Chibuzo Nwabufo Okwuosa, Jung-Woo Hwang, Jang-Wook Hur

**Affiliations:** Department of Mechanical Engineering (Department of Aeronautics, Mechanical and Electronic Convergence Engineering), Kumoh National Institute of Technology, 61 Daehak-ro, Gumi-si 39177, Gyeonsangbuk-do, Republic of Korea; 20246080@kumoh.ac.kr (Y.-N.J.); okwuosachibuzo3@kumoh.ac.kr (C.N.O.); jwhwang@kumoh.ac.kr (J.-W.H.)

**Keywords:** scanning electron microscopy, energy-dispersive spectroscopy, phosphating, flow rate, uniformity, zinc phosphate

## Abstract

Uniformity in material coating is not only essential for ensuring durability and long-term reliability but also for reducing costs, optimizing resources, and maintaining high-quality standards in industrial applications. Zinc phosphate is notable for coating steel surfaces due to its excellent corrosion resistance and adhesion properties in various industries. This study investigates the optimal flow rate of a diaphragm pump for achieving the effective and uniform coating of a steel cylinder. The coating uniformity was assessed using Scanning Electron Microscopy (SEM), focusing on layer thickness and elemental composition. A range of flow rates was analyzed to determine their influence on coating quality and regularity, with Energy-Dispersive Spectroscopy (EDS) revealing the distribution and homogeneity of the applied layer. The results identified a flow rate of 30 L/min as optimal with a thickness of 3.6 µm of coating on both sample sides, as it minimized surface defects and ensured consistent thickness across the cylinder. This study provides valuable insights for optimizing industrial coating processes, contributing to improved efficiency and reduced resource waste.

## 1. Introduction

In metal finishing, coating plays a crucial role in preventing environmental degradation, reducing wear, friction, and thermal degradation in high-stress environments, particularly in ferrous and non-ferrous metals. Ferrous metals, due to their high iron content, are highly susceptible to oxidation, leading to rust formation [1,2]. Although non-ferrous metals are not as susceptible to oxidation as ferrous metals, they can still undergo oxidation and chemical degradation [3,4]. Hence, material coatings are essential as they create barriers that inhibit the penetration of oxygen and moisture, thereby reducing corrosion. Among the most popular metal finishing techniques employed over time, phosphating is one of the most commonly used metal finishing techniques, not only because of its high corrosion resistance but also due to its improved structural abrasive resistance, good adhesion, and low manufacturing cost [5,6,7,8,9,10].

The phosphating process is fundamentally based on the electrochemical dissolution of the metal substrate and the simultaneous precipitation of metal phosphate layers. In its pure form, phosphating is referred to as iron phosphating, resulting in the formation of an iron phosphate (${\mathrm{FePO}}_{4}$) layer [11,12]. The process is summarized in Equations (1)–(4).

(1) $$H_{3}{\mathrm{PO}}_{4}\to 3H^{+}+{\mathrm{PO}}_{4}^{3-}$$

(2) $$\mathrm{Fe}(s)\to {\mathrm{Fe}}^{2+}(aq)+2e^{-}$$

(3) $$2H^{+}(aq)+2e^{-}\to H_{2}(g)$$

(4) $${\mathrm{Fe}}^{2+}(aq)+{\mathrm{PO}}_{4}^{3-}(aq)\to {\mathrm{FePO}}_{4}(s)$$

The phosphating process occurs through a combination of anodic and cathodic reactions. Iron in the substrate undergoes oxidation to release ${\mathrm{Fe}}^{2+}$ ions, as shown in Equation (2). Concurrently, hydrogen ions from the phosphoric acid are reduced to form hydrogen gas, as illustrated in Equation (3). The ${\mathrm{Fe}}^{2+}$ ions then react with phosphate ions, leading to the precipitation of iron phosphate, as shown in Equation (4). This phosphate layer provides basic corrosion resistance and enhances adhesion properties.

Though phosphating can generally be employed for corrosion resistance, surface preparation, and wear protection, specific types such as zinc phosphate, manganese phosphate, and nickel phosphate are utilized for tasks where they excel uniquely [7,13,14,15,16,17]. For instance, in studies and practical applications, zinc phosphate is best implemented for automotive parts and industrial equipment due to its high corrosion resistance and paint adhesion properties, low toxicity, and thermal stability [17,18,19,20,21]. Manganese phosphate, with its excellent wear resistance and friction reduction capabilities, is most suitable for coating engine components, firearms, gears, and heavy machinery [22,23,24,25]. Nickel phosphate, owing to its unique hardness and chemical resistance properties, is utilized in aerospace, electronics, and chemical processing equipment [26,27,28]. The general mechanism involved in these types of phosphate was summarized by Yan et al. in [29] and is presented in Equations (5)–(8).

(5) $$\mathrm{Fe}\to {\mathrm{Fe}}^{2+}+2e^{-}$$

(6) $$H^{+}+2e^{-}\to H_{2}↑$$

(7) $${\mathrm{HPO}}_{4}^{2-}+{\mathrm{Me}}^{2+}+yH_{2}O\to {\mathrm{MeHPO}}_{4}\cdot yH_{2}O$$

(8) $$2{\mathrm{PO}}_{4}^{3-}+x{\mathrm{Me}}^{2+}+yH_{2}O\to {\mathrm{Me}}_{x}{\left({\mathrm{PO}}_{4}\right)}_{2}\cdot yH_{2}O$$

${\mathrm{Me}}^{2+}$ stands for metal cations, ${\mathrm{Zn}}^{2+}$, ${\mathrm{Mn}}^{2+}$, and ${\mathrm{Ni}}^{2+}$.

Over time, phosphating techniques have been optimized to ensure efficiency and effectiveness in most applications. For instance, the composition of the phosphate bath has a significant impact on the properties of the resulting coatings. The authors in [30] stated that the addition of sodium molybdate to a phosphate bath positively affects the corrosion resistance of the coated layer. In their assessment, they discovered that an increase in sodium molybdate content in the bath decreases the corrosion current. In another study [31], Amadeh and Fouladi investigated the effect of temperature on the thickness and sludge weight of phosphate coatings. They utilized magnesium phosphate as the phosphate coating in their analysis. In their findings, they stated that an increased temperature improves coating thickness and bath efficiency, with an optimal temperature benchmarked at 80 °C. The authors further emphasized that raising the temperature above 80 °C increases the crystal size and reduces the coating density. Another key point highlighted was the influence of phosphating time on the coating thickness and sludge weight. In their analysis, nucleation first formed at around 5–10 min, and an optimized dense and uniform coating was achieved at 20 min. However, exceeding the 20 min mark resulted in a decrease in uniformity, density, and corrosion resistance during the phosphating process, highlighting the importance of both temperature and exposure time in phosphating. Similarly, Cheng et al. [32] emphasized in their study the crucial role of bath temperature and immersion time in phosphating. They reported that optimal corrosion resistance in their bath formation was achieved at 80 °C with an immersion time of 10 min. Furthermore, they stressed that increasing the coating bath temperature above 80 °C resulted in a decrease in immersion time, primarily because the activation energy of the phosphating process is lower at higher temperatures. In a related study [33], the importance of pH concentration was highlighted. The authors investigated the microstructure, composition, and influence of pH value on the formation of micro-cracks and the corrosion behavior of phosphate conversion coatings on AZ91D. In their findings, within the range of pH values from 2.5 to 4.0, they discovered that, as the pH value of the solution increased, the thickness of the coatings also increased. However, the micro-crack width and its distribution area decreased. They concluded that, at a pH value of 4.0, the obtained coating film exhibited the best quality, the highest corrosion resistance, and the smallest micro-crack width and distribution area.

In all the studies presented thus far, the importance and critical roles of parameters such as temperature, pH, and immersion time in the efficiency of the phosphating process have been clearly demonstrated. Nonetheless, it is important to note that these findings are largely based on empirical observations and are not yet grounded in a consistent theoretical framework, as highlighted in the review study [34]. For instance, the studies in [31,32] both reported that an optimal bath temperature of 80 °C yielded the best results, whereas the study in [35] found that a coated sample treated at 55 °C exhibited the best corrosion resistance. Furthermore, in a more recent investigation [36], the authors demonstrated that phosphating baths maintained at pH 2.44 and temperatures between 60 and 65 °C produced thicker films with higher zinc content and enhanced corrosion resistance. These discrepancies suggest that either certain factors need to be standardized to establish a universally accepted fundamental value or that some parameters are interdependent, meaning that variations in one parameter may influence the optimal temperature required to achieve the best coating performance. Therefore, a more systematic approach, integrating both empirical observations and fundamental theoretical analysis, is necessary to fully understand and optimize the phosphating process.

On a brighter note, the key takeaway from most of these studies is that, while some observations have been generally verified, they do not necessarily establish a universal paradigm. For instance, the studies highlighted that, although elevated temperatures may enhance corrosion resistance and promote uniform coating, excessively high temperatures can result in phosphate precipitation in the bath and the formation of pores and cracks on the coated surfaces [34,37]. However, the effects of certain parameters—particularly flow rate—on coating uniformity still remain insufficiently addressed, to the best of the author’s knowledge at the time this study was conducted. Though a recent study has revealed that factors such as the incorporation of modified boron nitride nanosheets and the optimization of bath thermal conditions can significantly influence coating uniformity [38], the role of flow dynamics remains largely inconclusive. Therefore, further investigation is required to establish broader generalizations regarding phosphating process parameters and their effects. Among the various techniques employed to assess coating texture, uniformity, and corrosion resistance, Scanning Electron Microscopy (SEM), X-ray Diffraction (XRD), and Energy-Dispersive X-ray Spectroscopy (EDS) remain the most widely used, as reported in several studies [5,15,31].

This study aims to uniquely investigate the influence of flow rate on the uniformity of zinc phosphate coatings by varying flow conditions while maintaining constant concentration and temperature—an aspect rarely addressed in previous studies. A real-time flow monitoring and regulation system was implemented using a diaphragm pump and flow-rate sensor, allowing precise control over the dynamic coating environment. This novel integration enables a deeper understanding of how flow behavior affects coating uniformity, which is critical for improving adhesion and corrosion resistance. By providing insights into optimal flow parameters, this work advances phosphating technology toward more efficient, consistent, and resource-conscious industrial coating applications.

## 2. Materials and Methods

### 2.1. Material Description

The steel sample used in this study is SWRCH45K, a medium carbon steel with a carbon content of approximately 0.45%, specifically designed for cold heading and forging applications. The designation SWRCH45K refers to Steel Wire Rod for Cold Heading, where 45 indicates the carbon content and K signifies that it is a killed steel. Killed steel is deoxidized during manufacturing using agents such as ferrosilicon, aluminum, or manganese, preventing gas emissions during solidification and resulting in a denser, more uniform microstructure with minimal segregation and defects. This process enhances the steel’s mechanical properties, including superior ductility, toughness, and high-temperature strength, making it ideal for demanding industrial applications. Additionally, the reduced oxygen content minimizes porosity formation during welding, improving workability and making it suitable for welding-critical components in industries such as automotive and aerospace. The chemical composition and mechanical properties of SWRCH45K is detailed in Table 1 and Table 2, respectively, based on KS D 3592 standards [39]. This steel conforms to the JIS G 3507 standard (Japanese Industrial Standard) [40] and is commonly produced by manufacturers such as POSCO (Pohang-si, Republic of Korea), Nippon Steel (Chiyoda-ku, Tokyo, Japan), and JFE Steel (Chiyoda-ku, Tokyo, Japan).

**Figure 1:**
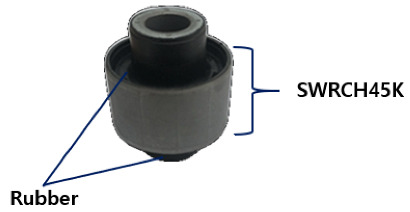
Coupler composed of rubber and SWRCH45K steel.

The material used in this study is part of a component known as a coupler, which is used in the C-EPS (Column-type Electric Power Steering) system and manufactured by TSR (Taesung Rubber & Chemical, Gumi-si, Republic of Korea). A diagrammatic representation of the coupler is shown in Figure 1. The coupler, a critical component of the steering motor shaft, transmits power within the steering system while absorbing shocks generated during steering. It is composed of rubber and additional materials, produced through a vulcanization process that enhances the elasticity and durability of the rubber. The C-EPS system operates by directly rotating the steering column using a steering motor when the driver turns the wheel, thereby adjusting the vehicle’s direction.

**Figure 2:**
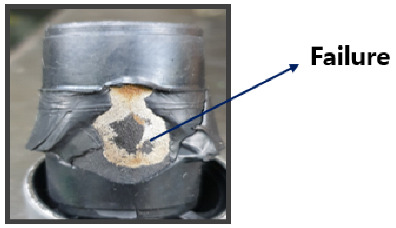
Adhesion failure in a poor coating instance.

The coupler is typically composed of a composite structure of steel (SWRCH45K) and rubber, with a phosphate coating treatment applied to the steel surface to promote effective adhesion. Structurally, the coupler consists of multiple layers, including rubber, adhesive, primer, a phosphate coating layer, and the steel substrate (SWRCH45K). The quality of the phosphate coating plays a critical role in ensuring bonding integrity; poor coating quality can result in adhesion failures [41,42]. As illustrated in Figure 2, such failures may manifest as rubber detachment from the metal surface. The phosphate layer not only provides corrosion resistance but also serves as a key interface for adhesive bonding. When the coating is uneven, discontinuous, or excessively thin, it can lead to insufficient adhesive coverage, compromising the bonding strength and leaving areas of the steel surface vulnerable to corrosion.

**Figure 3:**
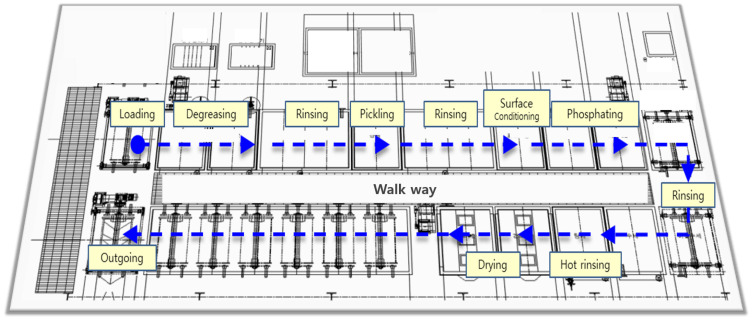
Multi-stage pre-treatment and pre-phosphating process layout.

The SWCH45K steel undergoes a multi-stage pre-treatment and pre-phosphating process to ensure optimal surface activation and coating formation as summarized in Figure 3. The process begins with degreasing, where two sequential steps are performed using Grapefruit Seed Extract (GSE) Ultra Cleaner, a powerful antimicrobial agent composed of grapefruit extract and glycerin. This step effectively removes oil, dust, and impurities from the steel surface, ensuring the quality of subsequent processes. The degreasing solution is maintained at an alkalinity of 45–50 mL, with a treatment temperature of 60–80 °C and a total processing time of 420±30 s. The concentration and condition of the degreasing solution are monitored using the neutralization titration method, checked three times per shift, and adjusted through chemical replenishment or dilution as needed. Temperature is monitored every four hours and regulated via a heating system to maintain the optimal range.

Following degreasing, rinsing is performed to remove residual chemicals. This step is critical to prevent coating defects, corrosion, or contamination in subsequent processes. Five rinsing steps (Rinse #1 to Rinse #5) are carried out, each lasting 30–80 s. The treatment time is automatically controlled by a timer to ensure consistency, and the rinse water is periodically replaced and continuously supplied to maintain quality. Filters are used to remove impurities, and the pH level of the rinse water is monitored every four hours to ensure it remains within the appropriate range.

Next, the pickling process is conducted using SK-LX#110, an acid-cleaning agent that removes rust, oxides, and impurities from the steel surface. This step activates the surface, ensuring uniform phosphate coating formation and enhancing corrosion resistance. The acid concentration is maintained at 20–25 mL, with a treatment time of 300±30 s. The pH and acid concentration are measured periodically, and the neutralization titration method is applied three times per shift. Residual acid is thoroughly removed through Rinse #3 and Rinse #4 to prevent unwanted reactions during phosphating. The surface conditioning process is then performed using D-Z solution, which activates the surface and facilitates the formation of fine crystalline nuclei. This step ensures a uniform phosphate coating with improved adhesion and a crystalline structure. The alkalinity of the solution is maintained at 1.5–3.0 mL, and the pH level is kept between 8.5 and 10.5. The treatment time is set to 210±30 s, and the chemical concentration is monitored three times per shift to prevent coating defects.

The phosphating process is the core step in forming a zinc phosphate film on the steel surface, enhancing corrosion resistance and improving adhesion strength for subsequent coatings. The chemicals used include Phosphating Agent #153 (a blend of zinc oxide, nitric acid, phosphoric acid, nickel carbonate, sodium chlorate, calcium nitrate, and citric acid monohydrate), Accelerator #138 (sodium nitrite, sodium carbonate, and purified water), and Neutralizer #405 (sodium hydroxide and sodium carbonate). Their respective concentrations are summarized in Table 3. To ensure a high-quality and uniform coating, the process conditions are carefully optimized: Total acidity is maintained at 34–38 mL to sustain the availability of reactive phosphate-forming ions, promoting effective nucleation. Free acidity is controlled between 2.5 and 4.5 mL to moderate the bath aggressiveness—preventing excessive metal dissolution or incomplete coating formation. The bath temperature is kept within 60–80 °C, enhancing reaction kinetics and supporting uniform crystal growth without introducing defects. A treatment duration of 420±30 s is selected to allow for adequate film development while minimizing overgrowth or brittleness. Following phosphating, hot rinsing at 80–100 °C for 120–150 s is conducted to remove residual chemicals and stabilize the surface morphology. The cleanliness and temperature of the rinse water are continuously monitored to prevent contamination and ensure surface integrity. X-ray Fluorescence (XRF) is employed for thickness verification, with inspections carried out three times per shift to maintain process consistency.

Finally, the drying process removes residual moisture, completing surface stabilization. This step is crucial for preventing corrosion and ensuring the quality of subsequent processes such as painting or assembly. The drying temperature is maintained at 155–195 °C, with a treatment time of 200–230 s. The dryer’s temperature settings are checked every four hours to ensure optimal conditions. Through this comprehensive pre-treatment and phosphating process, surface activation is achieved, ensuring the formation of a uniform and high-quality phosphate coating that enhances adhesion, corrosion resistance, and overall performance.

### 2.2. Zinc Phosphate Coating Process Details

**Figure 4:**
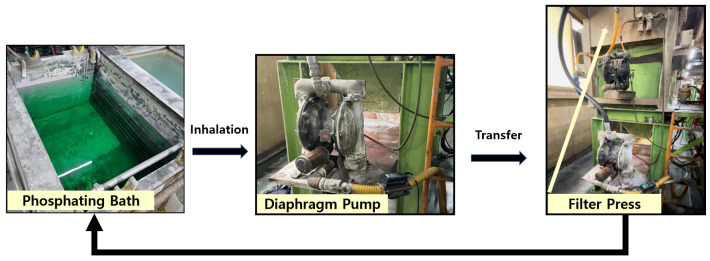
Zinc Phosphate Film Coating Process Using a Diaphragm Pump.

The phosphate coating process is a critical step in ensuring the formation of a uniform and high-quality coating on subcomponents. In this study, the process involves transferring subcomponents placed in a barrel system into a phosphate solution bath, where the coating is formed. To achieve consistent solution concentration and uniform coating formation, the phosphate solution is continuously circulated throughout the system. This circulation process is illustrated in Figure 4. The process begins with the phosphate solution being transferred from the bath using an air diaphragm pump, which ensures a steady flow of the solution. The solution then passes through a filter press, where any sludge or impurities are separated and removed. The filtered solution is subsequently recirculated back into the phosphate bath, maintaining the solution’s quality and consistency.

**Figure 5:**
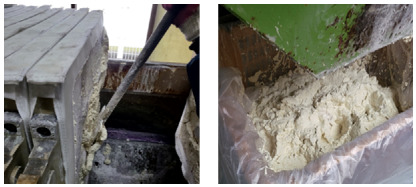
Sludge collection during the phosphating process.

The sludge separated by the filter press was continuously collected and discarded into a lower waste container, as depicted in Figure 5. This sludge management process was essential not only for preventing the accumulation of impurities in the phosphate bath but also for maintaining its chemical stability. Continuous circulation of the solution using a pump, along with in-line filtration via the filter press, ensured that impurities were effectively removed and did not accumulate in the bath. Maintaining a stable bath composition helped prevent inconsistencies in coating thickness, composition, and surface quality, which are commonly caused by sludge buildup and sedimentation. Furthermore, the pH and concentration of the bath were routinely monitored and adjusted throughout the experimental period to ensure the uniformity and reproducibility of the coating process.

In this study, particular attention was given to analyzing flow-rate variability, as it is a key factor influencing the uniformity of the phosphate coating. To address this, flow sensors were installed at the inlet and outlet of the air diaphragm pump. These sensors enable real-time monitoring and control of the flow rate, ensuring that the solution circulates at a consistent and optimal rate. By maintaining a stable flow rate, the study aims to achieve a uniform phosphate coating with consistent thickness and composition, ultimately enhancing the quality and performance of the coated components. This approach not only improves the reliability of the coating process but also contributes to the overall efficiency and effectiveness of the phosphating treatment.

**Figure 6:**
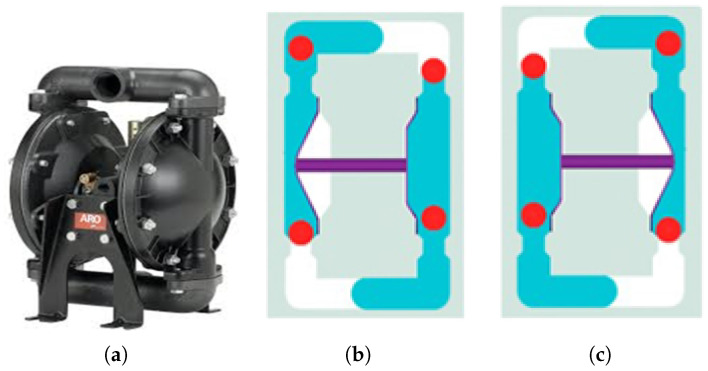
The diaphragm and its movement: (**a**) The diaphragm; (**b**) movement to the left; (**c**) movement to the right.

The air diaphragm pump used in this process, manufactured by ARO, is illustrated in Figure 6a. This pump utilizes compressed air to transport fluids and operates with two diaphragms and an air valve system. As shown in Figure 6b, the left diaphragm pushes into the fluid chamber, forcing liquid through the discharge port while the right diaphragm draws liquid into the right chamber. Upon stroke completion (Figure 6c), the diaphragms reverse positions, and the cycle repeats in the opposite direction. This alternating mechanism ensures a continuous and efficient flow of the phosphate solution, enhancing the stability and consistency of the coating process.

#### Flow-Rate Sensor Functionality and Setup

The air diaphragm pump plays a critical role in transporting the phosphate solution from the phosphate bath to the filter press. However, excessive sludge accumulation can reduce the pump’s suction power, leading to a decrease in flow rate, which negatively impacts the speed and uniformity of coating formation. To address this, real-time flow monitoring using flow sensors is essential to detect flow-rate reductions caused by sludge accumulation in advance. By analyzing flow data from the air diaphragm pump, this study aims to optimize process conditions, ensuring consistent coating quality and process efficiency.

**Figure 7:**
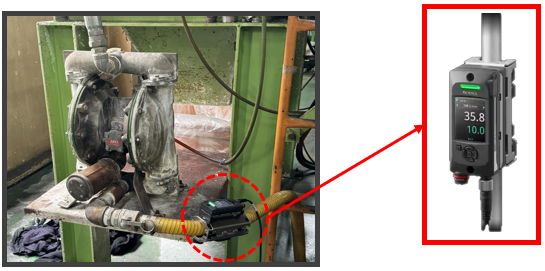
Flow-rate sensor attached to a pump.

**Figure 8:**
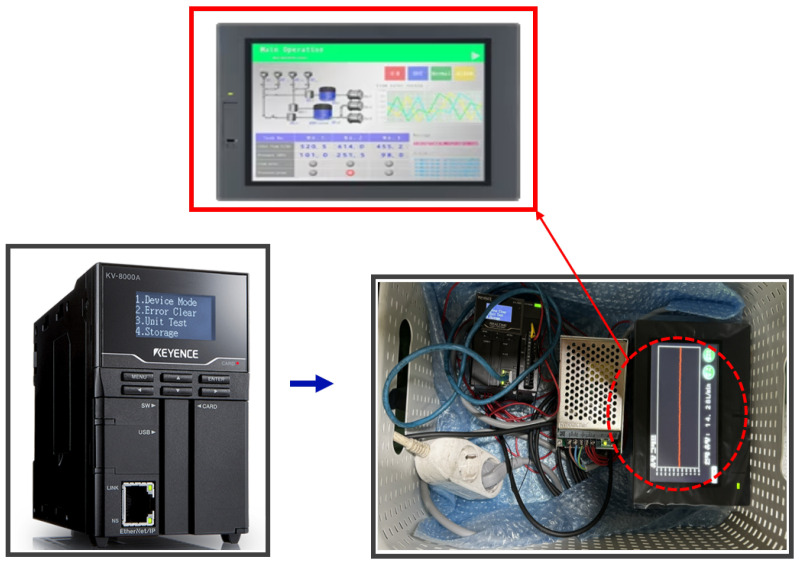
Monitor and display.

To measure and analyze flow-rate changes in real-time, a Keyence clamp-on flow sensor (FD-H series) was installed at the inlet and outlet of the air diaphragm pump, as shown in Figure 7. This non-contact sensor measures flow within the pipe and offers advantages such as ease of installation and low maintenance costs. The flow sensor data were collected and analyzed using a Keyence KV-8000 series Programmable Logic Controller (PLC), as shown in Figure 8. The PLC enables real-time data logging, continuously monitoring the set flow range. Additionally, the data were displayed on a Keyence VT5 series touch panel, as seen in Figure 8, allowing operators to visually monitor flow-rate variations and make timely adjustments to maintain optimal process conditions.

**Figure 9:**
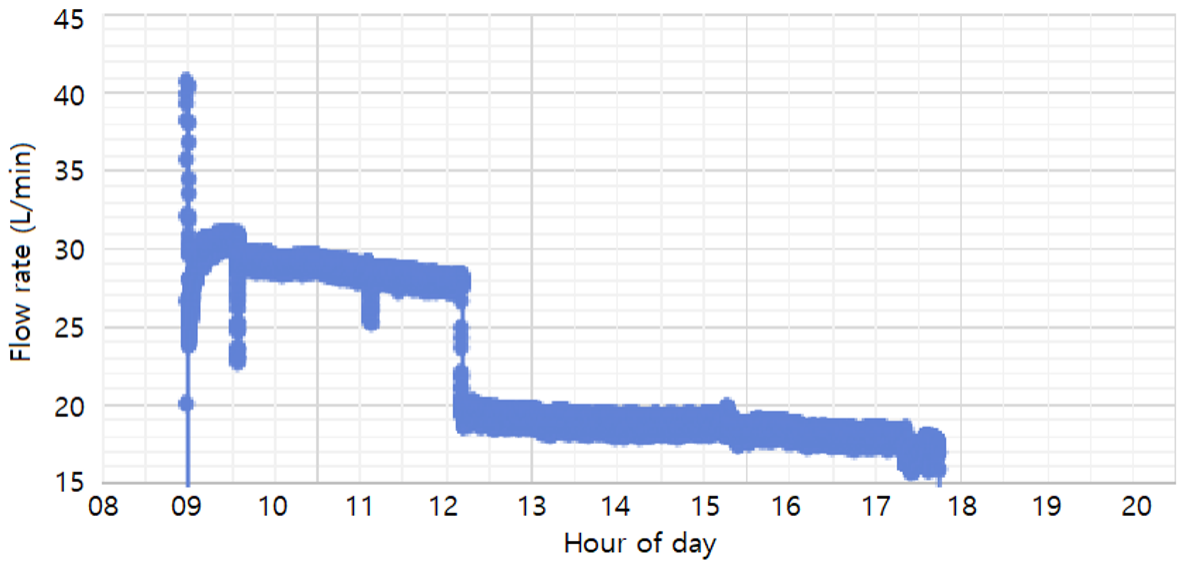
Flow rate during the phosphating process.

The flow rates used in the study (30 L/min, 20 L/min, and 10 L/min) were achieved by adjusting the float through the flow valve and monitored using a flow-rate sensor. As shown in Figure 9, the monitored flow rates during the process were maintained at 20 L/min and 30 L/min, with an additional 10 L/min condition included for experimental purposes. The 30 L/min and 20 L/min conditions were set as normal process conditions, ensuring uniform coating formation and smooth solution circulation. The 10 L/min condition was introduced as an extreme scenario, simulating significant sludge accumulation, to analyze its impact on coating thickness and uniformity. Based on these flow-rate conditions, the study compared coating thickness and uniformity across the different rates to determine the optimal flow rate for improving phosphate coating quality and process efficiency.

### 2.3. Specimen Preparation Process

**Figure 10:**
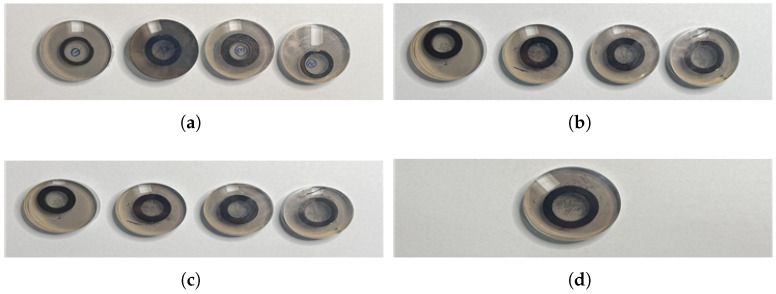
Specimens: (**a**) flow rate 10 L/min; (**b**) flow rate 20 L/min; (**c**) flow rate 30 L/min; (**d**) uncoated.

Specimen preparation began at the start of the phosphate coating process, with concentration and temperature held constant while flow rate was varied. Four specimens were prepared at 30 L/min, with each specimen processed at 15 min intervals. The same procedure was repeated for 20 L/min and 10 L/min, producing four specimens for each flow rate, resulting in a total of 12 phosphate-coated specimens. Additionally, untreated control specimens were prepared for comparison. the specimens employed in the study are shown in Figure 10.

**Figure 11:**
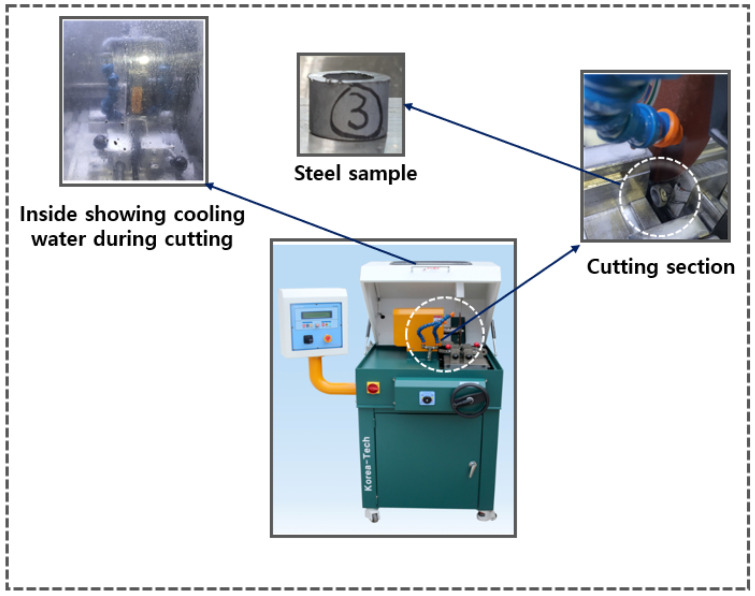
AMC-254 DUAL precision specimen cutter used for sample cutting.

For specimen cutting, the AMC-254 DUAL precision specimen cutter (KoreaTech Co., Ltd., Busan, Republic of Korea) was used, as shown in Figure 11. This equipment is equipped with a screw-driven vise system that allows forward and backward movement, along with a cam-type quick-release vise (left/right) to securely hold the specimen and minimize vibration during cutting. The feeding mechanism supports both automatic and manual operation, offering flexibility depending on the material. The specimen, a coupler made of SWCH45K steel, had an outer diameter of 20 mm, an inner diameter of 15 mm, and a height of approximately 15 mm. It was sectioned using a precision cutting wheel with an outer diameter of 254 mm, a thickness of 1.2 mm, and an arbor hole diameter of 31.75 mm. These specifications ensured an accurate cross-section while minimizing deformation during the cutting process. To avoid excessive heat generation and surface damage, an erosion-based cooling system was employed, circulating 60 L of cooling water and maintaining a constant temperature. The cutter, equipped with two 100 W motors, enabled stable cutting within a 450 × 580 × 430 mm range, as specified by the manufacturer. The cutting process can be seen in Figure 11.

Mounting, the process of fixing the specimen to facilitate handling, was performed to stabilize the specimen and prevent deformation during grinding and analysis, particularly for small or irregularly shaped specimens. This process ensures even surface processing, maintains consistent shape, and enhances reproducibility. The hot compression mounting method was employed using a Mounting Press M/C machine, set to an initial temperature of 17 °C, a target temperature of 149 °C, a holding time of 10 min, and a cooling time of 16 min. The process rate was set at 74.1% to ensure uniform resin distribution. Transparent Thermoplastic Powder from Allied High Tech Products Inc. was used as the mounting resin due to its transparency, durability, and resistance to friction, making it suitable for microscale observation using SEM. Before mounting, the specimen was thoroughly cleaned to remove contaminants, placed in the mounting chamber, and encased in resin under heat and pressure. After cooling, the mounted specimen was prepared for grinding.

**Figure 12:**
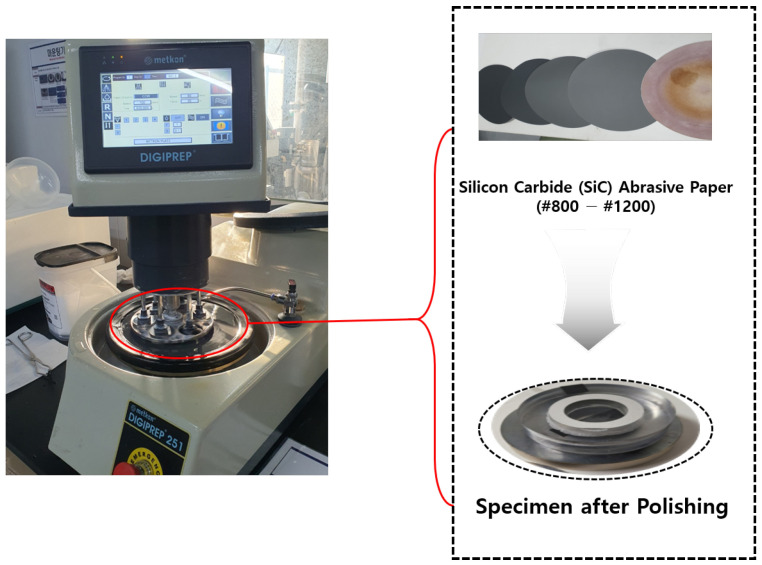
Metkon Digirep 251 used for sample grinding and polishing.

Grinding and polishing were conducted to flatten the specimen surface, remove irregularities, and achieve a smooth, reflective finish for SEM analysis. The Metkon DIGIPREP 251 machine, as shown in Figure 12, was used for both processes. Grinding involved the stepwise use of Silicon Carbide (SiC) abrasive paper: coarse grinding with SiC #320 → #600 to remove mounting irregularities, followed by fine grinding with SiC #800 → #1200. The rotation speed was adjusted from 110 r/min (620 s) to 80 r/min, with a grinding force of 35 N. Polishing utilized diamond suspension (6 µm → 3 µm) on a diamond polishing pad and colloidal silica (0.05 µm) on a nap cloth or velvet pad to achieve a smooth surface. The platen rotation direction (CCW) and polishing speed (80 r/min) were maintained, with a pressure of 35N applied throughout.

**Figure 13:**
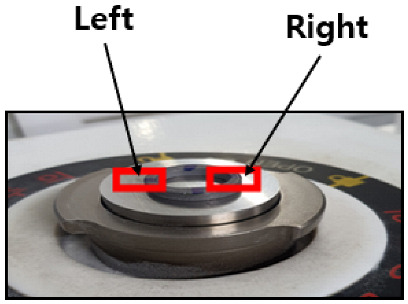
SEM and EDS measurement locations.

Each donut-shaped specimen was analyzed via EDS and SEM on both left and right sides (Figure 13) to quantify coating uniformity and composition changes across flow variations. For each flow rate (10, 20, and 30 L/min), measurements were taken at four immersion intervals (0 min, 20 min, 40 min, and 60 min), yielding eight data points per flow rate (2 sides × 4 time points).

### 2.4. SEM and EDS Analysis Setup

Sample preparation involved addressing the poor conductivity of the specimen to prevent charging effects during electron beam exposure. The specimen was attached to a holder using conductive carbon tape, which provided a conductive path along the surface, minimizing charging artifacts. The surface charge density (σ) can be described as:

(9) $$\sigma =\frac{Q}{A}$$

where *Q* is the total charge and *A* is the surface area. The use of conductive materials helps dissipate charge, which can be further explained using Ohm’s Law:

(10) $$J=\sigma_{c}E$$

where *J* is the current density, $\sigma_{c}$ is the electrical conductivity, and *E* is the electric field.

To further enhance conductivity, platinum coating was performed using the MC1000 Ion Sputter Coater (Hitachi, Tokyo, Japan). The coating process involved creating a vacuum environment, placing the specimen 33 mm above the platinum target, setting the sputtering current to 15 mA, and coating for 60 s. After coating, the specimen was mounted onto the SEM holder for analysis.

SEM analysis was conducted using the FE-SEM 700HR (Hitachi, Japan). The specimen was mounted on the SEM holder and inserted into the loading chamber. After vacuum formation, the sample was moved into the SEM chamber, and parameters such as acceleration voltage (15 kV), working distance (10 mm), and imaging mode (Secondary Electron and Backscattered Electron) were set. The interaction of the electron beam with the specimen can be described using the electron penetration depth (R), approximated by the Kanaya–Okayama range equation:

(11) $$R=\frac{0.0276\cdot A\cdot E_{0}^{1.67}}{\rho \cdot Z^{0.89}}$$

where *A* is the atomic weight of the specimen and $E_{0}$ is the atomic number. The electron beam was activated, and contrast, brightness, and focus were adjusted to optimize the image.

EDS analysis was performed using the AZtec Energy Advanced Package, version 6.1 HF4 (Oxford Instruments, Abingdon, UK). The EDS detector was inserted, and line scan analysis was conducted along the coating layer to measure elemental distribution. The Energy-Dispersive X-ray Spectroscopy (EDS) process involves the emission of characteristic X-rays, which can be described using Moseley’s Law:

(12) $$\nu =k{\left(Z-\sigma \right)}^{2}$$

where ν is the frequency of the emitted X-ray, *K* is a proportionality constant, *Z* is the atomic number of the element, and σ is a shielding constant. This equation forms the basis for elemental identification in EDS. The electron beam voltage was set to 15 kV for optimal signal detection, and Pt/Zr (Platinum/Zirconium) coating elements were excluded from the final dataset.

## 3. Results and Discussion

**Figure 14:**
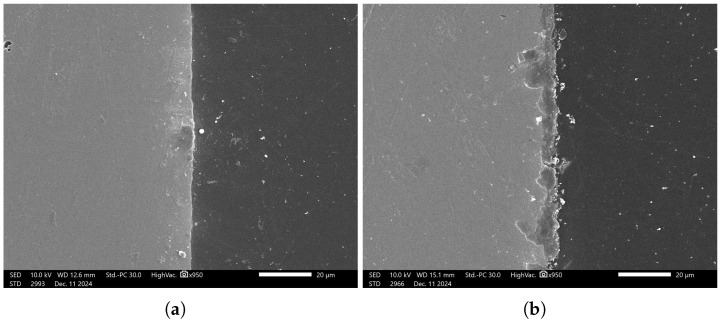
SEM images: (**a**) before phosphating; (**b**) after phosphating.

To address the study objectives, the samples were analyzed using Scanning Electron Microscopy (SEM) and Energy-Dispersive X-ray Spectroscopy (EDS) to characterize their microstructure and chemical composition. Figure 14 presents SEM micrograph sample of the coated specimens, demonstrating the presence of a phosphate coating layer.

**Figure 15:**
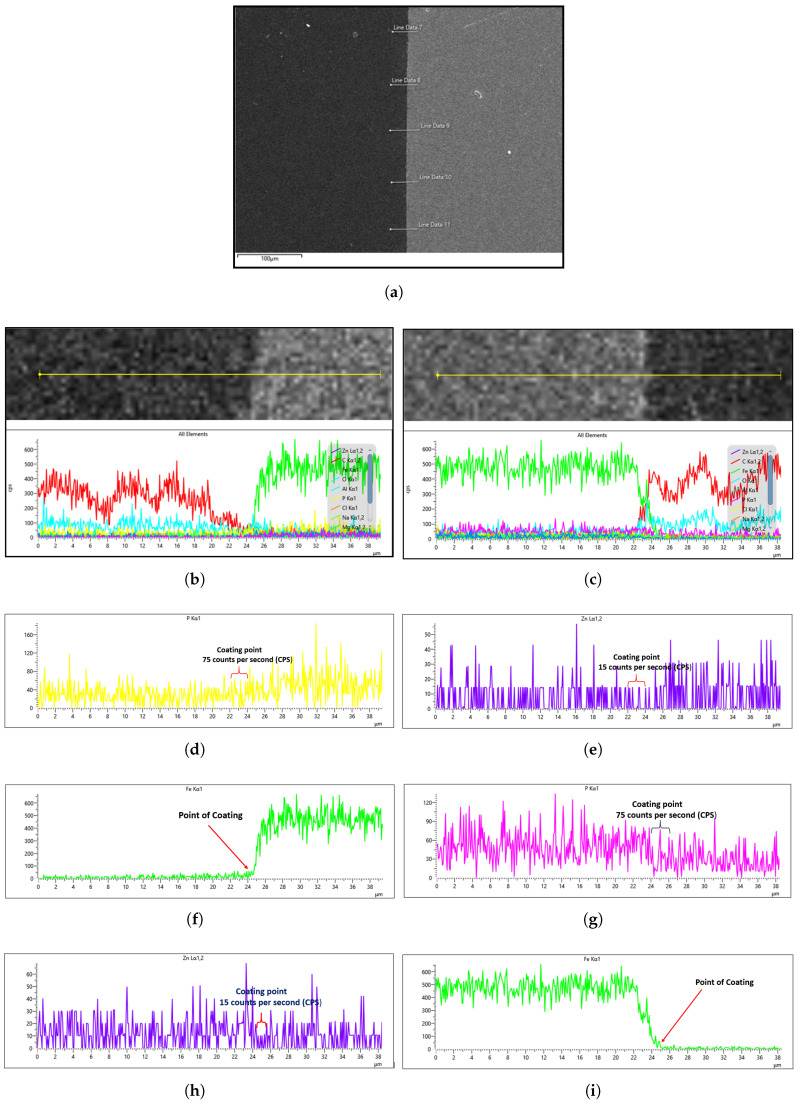
EDS of a sample before coating: (**a**) analysis spot; (**b**) EDS of all elements on the left side of the sample; (**c**) EDS of all elements on the right side of the sample; (**d**) EDS of P (**left** side); (**e**) EDS of Zn (**right** side); (**f**) EDS of Fe (**left** side); (**g**) EDS of P (**right** side); (**h**) EDS of Zn (**left** side); (**i**) EDS of Fe (**right** side).

To characterize the phosphate coating composition, EDS analysis was conducted. Prior to coated sample evaluation, the uncoated steel substrate was analyzed by EDS to establish a baseline reference. For each sample, five measurement points per side (Figure 15a) were assessed to account for potential surface irregularities introduced during mechanical preparation (e.g., abrasive paper polishing). The most representative spectrum, excluding regions with evident preparation artifacts, was selected for quantitative comparison.

The general EDS result of the uncoated steel for both the right and left sides of the sample is shown in Figure 15b,c. This cross-sectional analysis specifically focuses on the interface between the steel sample and the mounting platform (visible as distinct light gray and dark gray regions in Figure 15a), where the coating substance is clearly observable. To enhance the interpretation of the coating analysis, Fe EDS mapping is employed to monitor the presence of iron in the coated region. A drop in Fe concentration in the EDS signal indicates either the absence of iron in the coating or the suppression of its signal due to the phosphating process. Hence, the sharp decline in Fe concentration can be attributed to the fact that the platform has no Fe content, as the steel is yet to undergo phosphating, as shown in the EDS images in Figure 15f,i.

Since the phosphating agent was zinc phosphate, we specifically monitored the phosphorus (P) and zinc (Zn) concentrations to evaluate the coating degree. Figure 15d,e,g,h demonstrate that both the left and right sides of the uncoated sample showed similar P and Zn levels, establishing a reliable baseline for subsequent coated sample analysis. Notably, the uncoated steel exhibited higher trace phosphorus content than zinc content, as seen in Figure 15d,g, which is expected since SWRCH45K typically contains minor phosphorus impurities, as specified in the JIS G3507 standards.

**Figure 16:**
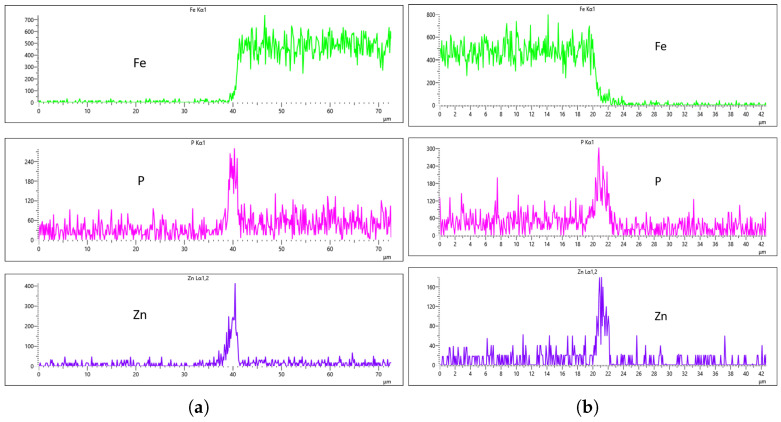
EDS plot for 10 L/min flow-rates: (**a**) at 0 min left; (**b**) at 0 min right.

**Figure 17:**
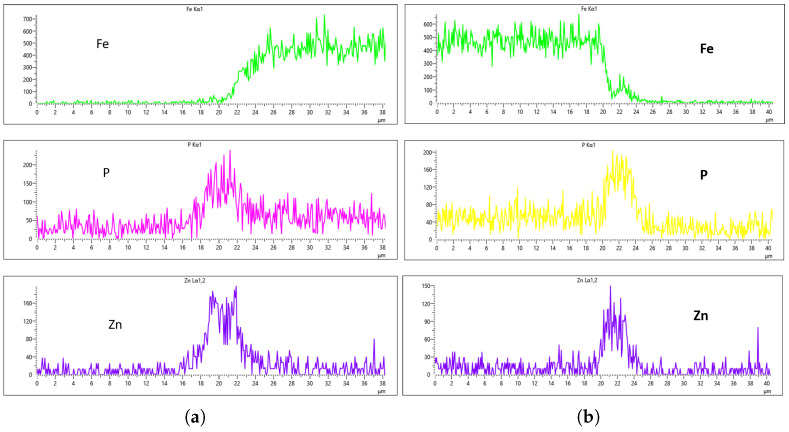
EDS plot for 10 L/min flow-rates: (**a**) at 20 min left; (**b**) at 20 min right.

**Figure 18:**
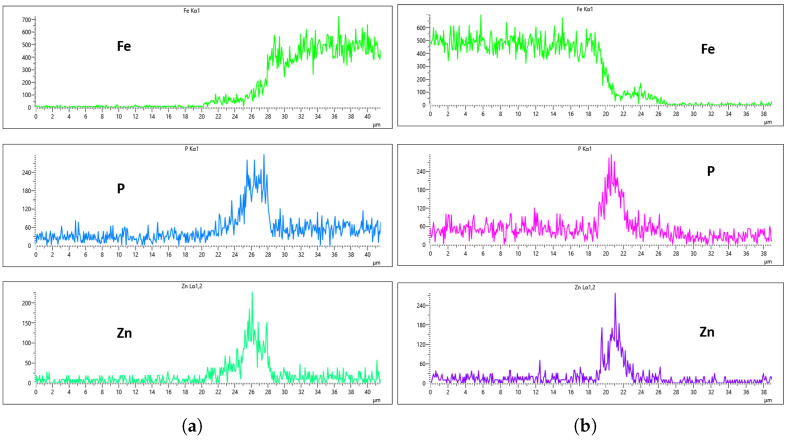
EDS plot for 10 L/min flow-rates: (**a**) at 40 min left; (**b**) at 40 min right.

**Figure 19:**
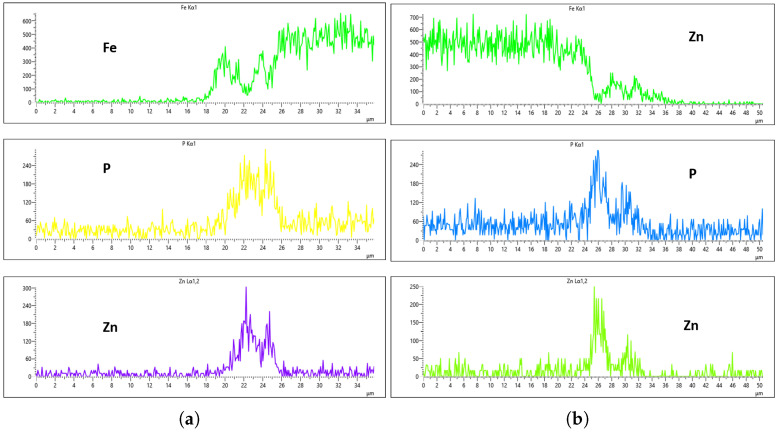
EDS plot for 10 L/min flow-rates: (**a**) at 60 min left; (**b**) at 60 min right.

As highlighted in the previous section, three flow rates were analyzed, 10 L/min, 20 L/min, and 30 L/min, with the aim of determining the optimal flow rate for uniformity. These flow rates were monitored at immersion intervals of 0 min, 20 min, 40 min, and 60 min. Figure 16, Figure 17, Figure 18 and Figure 19 presents the EDS results for 10 L/min across these four immersion time intervals. In addition to the primary elements monitored, Fe was included in the plots to assess how effectively the coating suppressed its presence on the surface. The results reveal notable differences across the immersion intervals. For example, in Figure 16a,b, Fe content was effectively suppressed, showing minimal presence on the coating surface. However, starting from Figure 17b, Fe concentration began to rise and became significantly higher in Figure 19a,b. Additionally, the P and Zn signals at 0 min of immersion (Figure 16a,b) appeared weaker and the coating thinner compared to later immersion times. As the immersion time increased, the coating thickness also increased, particularly at a flow rate of 10 L/min, as observed in Figure 17, Figure 18 and Figure 19. Furthermore, the rising Fe content with longer immersion times suggests possible Fe diffusion or contamination within the coating layer, potentially compromising the overall coating quality at this flow rate.

**Figure 20:**
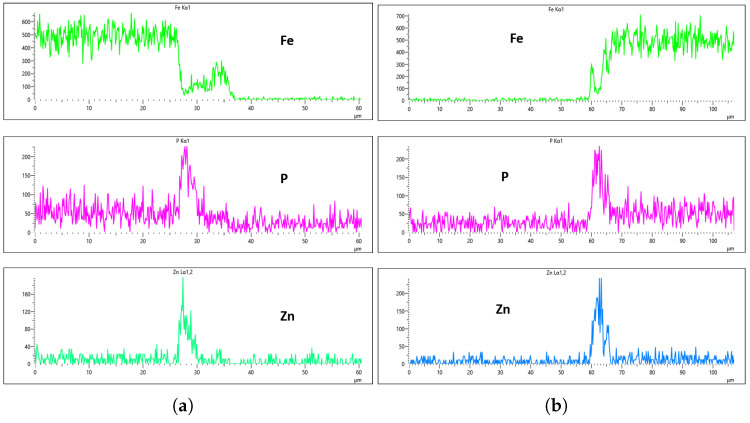
EDS plot for 20 L/min flow-rates: (**a**) at 0 min left; (**b**) at 0 min right.

**Figure 21:**
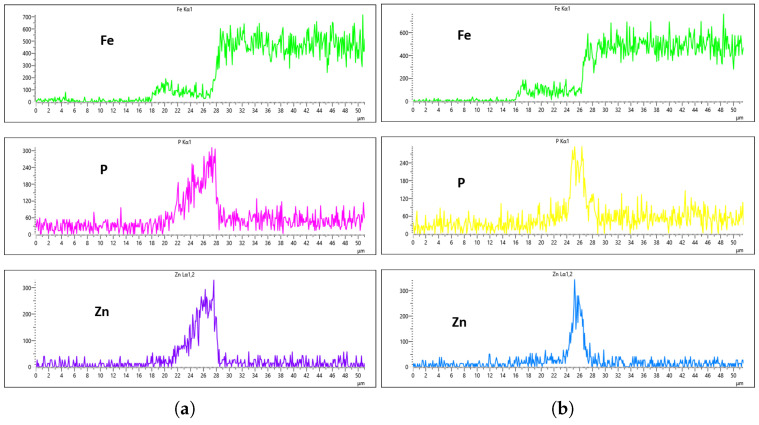
EDS plot for 20 L/min flow-rates: (**a**) at 20 min left; (**b**) at 20 min right.

**Figure 22:**
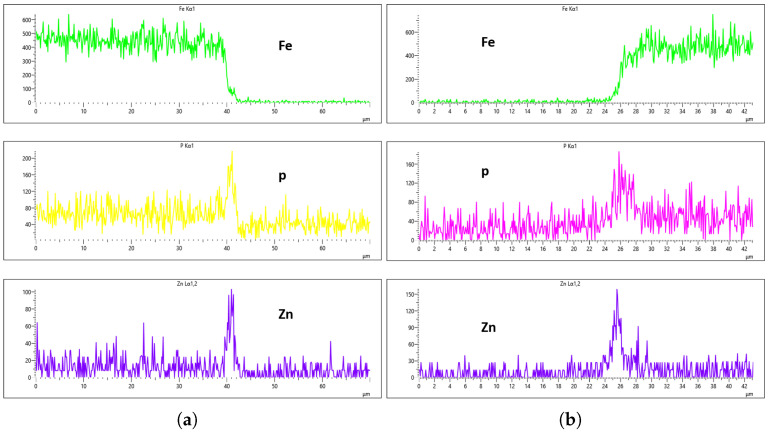
EDS plot for 20 L/min flow-rates: (**a**) at 40 min left; (**b**) at 40 min right.

**Figure 23:**
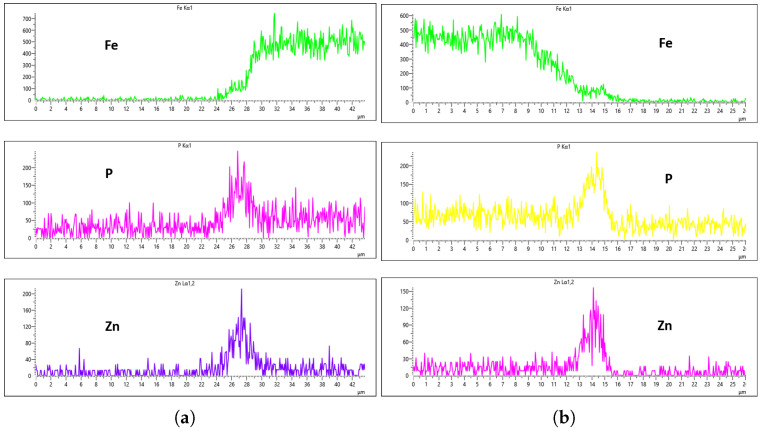
EDS plot for 20 L/min flow-rates: (**a**) at 60 min left; (**b**) at 60 min right.

Figure 20, Figure 21, Figure 22 and Figure 23 illustrate the EDS analysis results for samples coated at a flow rate of 20 L/min across different immersion time intervals. In contrast to the observations made at the 10 L/min flow rate, the coating formed at 0 min of immersion did not effectively suppress the Fe content within the coating layer, as clearly evidenced in Figure 20. Interestingly, at 40 min of immersion (Figure 22), the coating demonstrated a significant suppression of Fe content, indicating a well-developed coating layer with a more complete coverage of the steel substrate. However, this trend did not continue linearly with further immersion time. By 60 min of immersion, as seen in Figure 23, the Fe signal reappeared more prominently in the coating layer, suggesting a possible breakdown or thinning of the coating at prolonged exposure times, or the onset of Fe diffusion from the substrate into the upper layers of the coating. These observations imply that the optimal immersion time for achieving effective Fe suppression at a 20 L/min flow rate is approximately 40 min. It may also fall within a narrow window between 40 and 60 min, beyond which coating integrity might begin to deteriorate or allow Fe to resurface. Furthermore, the relationship between immersion time and coating thickness is evident. The coating layer at 0 min is noticeably thinner and less developed compared to those observed at extended immersion times, as shown in Figure 20 and Figure 21.

**Figure 24:**
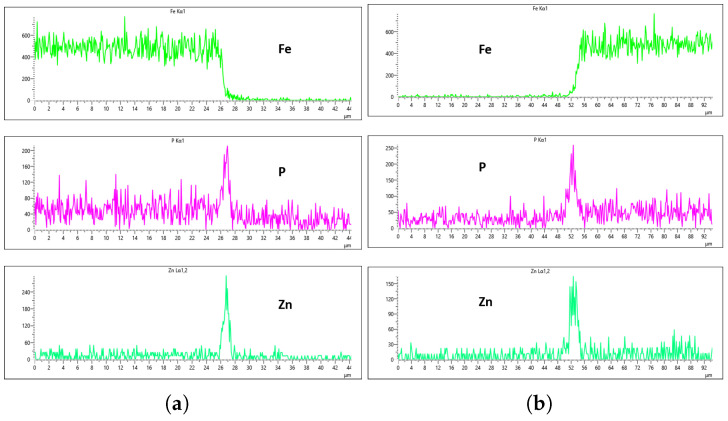
EDS plot for 30 L/min flow-rates: (**a**) at 0 min left; (**b**) at 0 min right.

**Figure 25:**
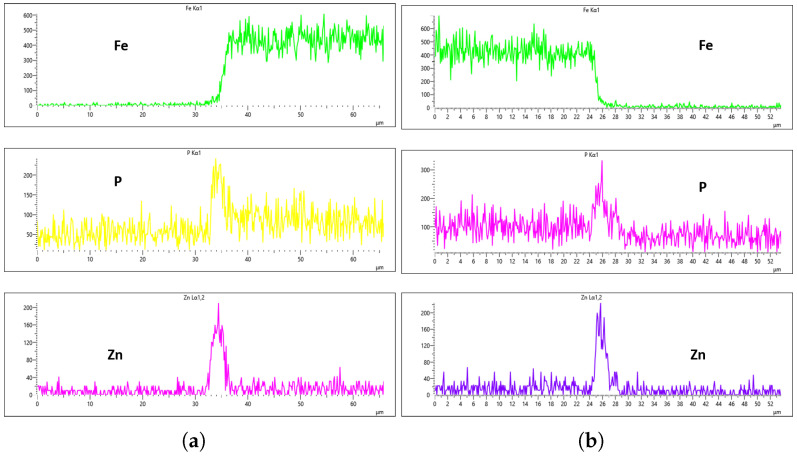
EDS plot for 30 L/min flow-rates: (**a**) at 20 min left; (**b**) at 20 min right.

**Figure 26:**
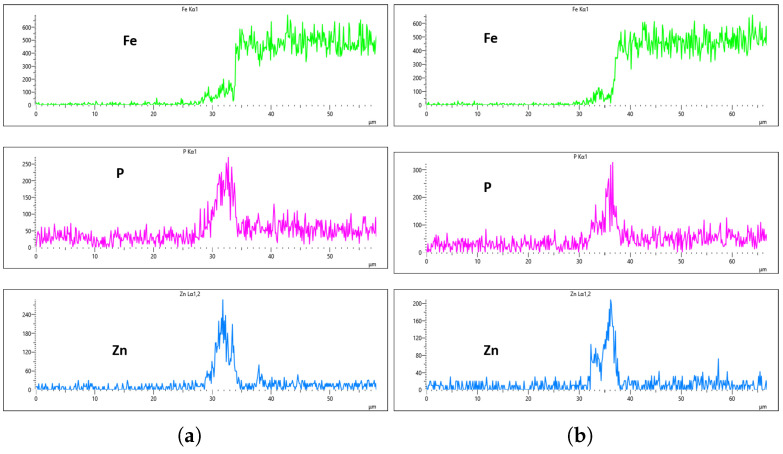
EDS plot for 30 L/min flow-rates: (**a**) at 40 min left; (**b**) at 40 min right.

**Figure 27:**
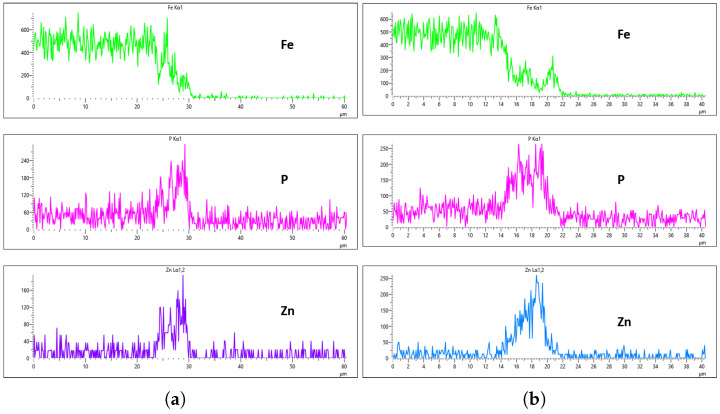
EDS plot for 30 L/min flow-rates: (**a**) at 60 min left; (**b**) at 60 min right.

Figure 24, Figure 25, Figure 26 and Figure 27 show EDS analysis conducted at a flow rate of 30 L/min across different immersion time intervals. The pattern observed here shares similarities with the results from the 10 L/min flow rate. Specifically, at 0 min of immersion, the coating effectively suppressed the presence of Fe on the surface, as illustrated in Figure 24a,b. This suggests that even a brief exposure to the zinc phosphate solution can initiate the formation of a protective coating capable of shielding the steel substrate. In contrast to the 10 L/min case, Fe suppression was also maintained at the 20 min immersion mark for 30 L/min. This extended suppression indicates that the higher flow rate may promote a more uniform or denser coating during the early stages of the treatment process, enhancing the barrier against Fe diffusion or detection. As the immersion time increased, the Fe signal began to appear within the coating layer. At 40 min, a slight presence of Fe was detected, implying possible thinning or localized disruption of the coating. By 60 min, as shown in Figure 26 and Figure 27, the Fe content became more pronounced, suggesting that prolonged immersion may lead to reduced coating efficiency, potentially due to microstructural changes or the onset of diffusion processes allowing Fe from the substrate to migrate upward. Regarding coating thickness, the layer formed at 0 min was the thinnest among all immersion durations, which aligns with observations at other flow rates.

### Result Discussion

In the context of this study’s primary objective—which is to determine the influence of flow rate on the uniformity and overall effectiveness of the zinc phosphating process—three fundamental factors were identified as critical in selecting the optimal flow rate for this particular bath composition. These include (i) the concentration of phosphorus (P) and zinc (Zn) on the coated surface, (ii) the presence and suppression level of iron (Fe) within the coating layer, and (iii) the resulting thickness of the applied coating film. Among the notable and consistent trends observed throughout all examined flow rates is the noticeably thinner coating formed at a 0 min immersion time. This trend is visually evident from the EDS plots, where the µm-scale spikes representing P and Zn signals along the x-axis are relatively low in magnitude and narrow in width, indicating a very light or minimal coating.

EDS results further confirm that both the immersion duration and the flow rate exert significant influence over the final coating thickness and composition. To identify the most effective flow rate for producing a uniform and high-quality coating, a detailed comparison was conducted across the EDS results for each flow rate and immersion interval. At a flow rate of 10 L/min, Fe was successfully suppressed at the 0 min immersion interval, but its suppression efficiency decreased with increased immersion time—being least effective at 60 min. Interestingly, despite the higher Fe presence, the 60 min immersion under 10 L/min flow rate exhibited the most uniform coating distribution between the left and right sections of the sample. As demonstrated in Figure 19a,b, the P concentration reached values of 296 Counts Per Second (CPS) and 282 CPS, respectively, while the Zn concentration was recorded at 300 CPS and 250 CPS for the same regions. These values corresponded to an average coating thickness of approximately 6 µm on both sides. This uniformity in thickness and elemental distribution is a positive outcome; however, the accompanying presence of Fe contamination within the coating—as shown in the same figures—raises concern about the potential impact on the corrosion resistance and structural integrity of the coating, making it a less-than-ideal condition.

For the 20 L/min flow-rate condition, the EDS analysis reveals a different behavior compared to the 10 L/min case. Specifically, Fe was present from the onset at the 0 min immersion and was gradually reduced until it was nearly or fully suppressed at 40 min of immersion. Unfortunately, this suppression did not persist, as Fe signals re-emerged slightly at the 60 min mark, as seen in Figure 23a,b. Although the results at the 40 min interval indicate some improvement in Fe suppression, the overall uniformity of the coating remained inconsistent. For instance, as depicted in Figure 22, the phosphorus concentration on the left and right surfaces of the sample were 215 CPS and 185 CPS, respectively. These corresponded to thickness values of 3 µm and 1.5 µm. Likewise, the Zn content measured at 110 CPS on one side and 160 CPS on the other side, translating to similar variations in coating thickness. These discrepancies, shown in Figure 22a,b, highlight the lack of uniform deposition at this flow rate, suggesting that, despite some positive trends, the 20 L/min condition still falls short of delivering a uniformly thick and well-composed zinc phosphate coating across the substrate surface.

In the case of the 30 L/min flow-rate EDS analysis, the presence of Fe was effectively suppressed at the 0 min immersion mark, similar to the trend observed for the 10 L/min flow rate, as illustrated in Figure 24. However, Fe signals began to reappear in the coating at 40 min of immersion and became more prominent at 60 min, indicating a gradual increase in Fe incorporation with extended immersion time. Interestingly, the overall results for the 30 L/min condition suggest that this flow rate generally yielded good coating quality and uniformity across most immersion durations. An exception was noted at the 0 min immersion interval, where a disparity in Zn concentration was observed between the left and right sides of the sample—specifically, the Zn CPS on the left side was lower than that on the right, as shown in Figure 24a,b.

Given that the presence of Fe in the coating is a critical criterion in determining the optimal flow rate, the 20 min immersion time stands out as the most favorable condition for the 30 L/min flow rate. At this immersion duration, Fe contamination was completely absent, and the concentrations of both P and Zn were nearly identical on both sides of the sample. The P concentration on the left and right regions was measured at 290 CPS and 320 CPS, respectively, corresponding to a uniform coating thickness of approximately 3.6 µm. Similarly, the Zn concentration reached 212 CPS on one side and 220 CPS on the other, also yielding a consistent thickness of 3.6 µm across the sample, as illustrated in Figure 25a,b. This high level of elemental symmetry and thickness consistency marks it as the most uniform coating result in this study.

To ensure that our assessments were not based solely on the graphical representations from the EDS plots, we also determined the atomic percentages of key elements such as iron (Fe), phosphorus (P), and zinc (Zn). These values are presented in Table 4, offering a more complete validation of the trends observed earlier and providing deeper insight into how both immersion time and flow rate influence the distribution of elements and the uniformity of the coating across the left and right sides of the samples.

Looking closely at the table, the data strongly support the previous analysis and highlights the significance of Fe suppression as a key indicator of effective phosphating. Among all the tested conditions, the sample treated at a 30 L/min flow rate for 20 min stood out as the most consistent and effective. In this condition, Fe content was significantly reduced, with values of 2.34% on the left side and 2.67% on the right, suggesting strong resistance to contamination and excellent surface coverage. The phosphorus concentrations were also nearly identical between sides, with 20.31% on the left and 20.92% on the right. Similarly, zinc values were closely aligned at 18.28% and 19.34%. These balanced results reflect a highly uniform and well-formed zinc phosphate coating.

In comparison, lower flow rates did not perform as well, even with the same immersion time. At 10 L/min, the Fe content rose to around 14 to 15%, indicating a weaker coating and more exposure of the base metal. The phosphorus and zinc values were also noticeably lower and less consistent between the two sides, averaging around 14.77% for P and 11% for Zn. The 20 L/min flow rate showed moderate results, with Fe at 8.22% on the right side, and somewhat better P and Zn values of around 16% and 13 to 15%, respectively, though not as balanced or uniform as the 30 L/min case. When it comes to uniformity, the best performance again came from the 30 L/min flow rate at 20 min. The minimal differences in atomic percentages between the left and right sides show that the coating was applied evenly. On the other hand, the worst uniformity appeared at 0 min immersion for both the 10 and 20 L/min conditions. For instance, at 20 L/min and 0 min, Fe was 11.99% on the left but jumped to 16.71% on the right, clearly indicating an uneven deposition.

One key trend that emerged from the data is the relationship between flow rate and required immersion time. Generally, higher flow rates required shorter immersion times to achieve good coating results. This pattern was especially clear with the 30 L/min flow rate, which provided the best outcome in just 20 min. In contrast, lower flow rates needed longer immersion times, but this also increased the risk of Fe contamination due to overexposure or degradation of the coating. Therefore, increasing the flow rate appears to be a more efficient and reliable strategy to ensure high-quality coating within a shorter period.

Overall, the atomic percentage data strongly supports the earlier findings from the EDS plots. It reinforces that the flow rate of 30 L/min combined with a 20 min immersion time produces the most effective and uniform zinc phosphate coating in this study. This setting offers the ideal balance of low Fe content, consistent P and Zn deposition, and reliable coating quality across both sides of the sample.

## 4. Conclusions

This study examined the effect of flow rate on the uniformity of zinc phosphate coatings on steel cylinders, utilizing a Scanning Electron Microscope (SEM) equipped with Energy-Dispersive Spectroscopy (EDS) to analyze coating thickness and elemental composition. The results demonstrated that both flow rate and immersion time significantly influence coating quality, with an inverse relationship observed between the two parameters. Among the tested flow rates (10 L/min, 20 L/min, and 30 L/min), a flow rate of 30 L/min with a 20 min immersion time was identified as optimal. This condition yielded a uniform coating thickness of 3.6 µm, with comparable CPS counts for both P and Zn, while minimizing Fe contamination. The study highlights that while higher flow rates reduce the required immersion time and improve coating uniformity, lower flow rates necessitate longer immersion times, increasing the risk of Fe incorporation, which can compromise corrosion resistance.

While this study successfully controlled key parameters such as temperature and bath concentration, one area that remains open for further investigation is surface roughness, which plays a crucial role in coating adhesion and overall performance. Although surface topography analysis was beyond the scope of the present research, future studies could explore the relationship between flow rate and surface roughness uniformity. Such an investigation would provide valuable insights into how flow conditions impact coating adhesion and long-term durability, thereby expanding the understanding of optimal phosphating parameters.

## Figures and Tables

**Table 1:** The chemical composition of SWRCH45K material.

Element	Weight Percent (wt.%)
Carbon	0.42–0.48
Silicon	0.1–0.35
Manganese	0.6–0.9
Phosphorus	max 0.03
Sulphur	max 0.035
Nickel	max 0.2
Chromium	max 0.2
Copper	max 0.3

**Table 2:** The mechanical properties of SWRCH45K material.

Property	Value
Tensile Strength (MPa)	780 max
Yield Strength (MPa)	590 max
Elongation (%)	10 max
Hardness (HBW)	180∼230

**Table 3:** Bath composition of the phosphating process.

Parameter	Chemical Used	Composition	Concentration
Total Acidity	Phosphating Agent #153	$H_{3}$ ${\mathrm{PO}}_{4}$	11 g/L
		${\mathrm{HNO}}_{3}$	4.32 g/L
		ZnO	8.1 g/L
Free Acidity	Neutralizer #405	NaOH	0.75 g/L
		${\mathrm{Na}}_{2}$ ${\mathrm{CO}}_{3}$	0.06 g/L
Acceleration	Accelerator #138	${\mathrm{NaNO}}_{3}$	0.26 g/L
		${\mathrm{Na}}_{2}$ ${\mathrm{CO}}_{3}$	0.12 g/L

**Table 4:** EDS elemental composition (at.%) at different immersion times and flow rates.

Flow Rate	0 min (%)			20 min (%)			40 min (%)			60 min (%)		
	Fe	P	Zn	Fe	P	Zn	Fe	P	Zn	Fe	P	Zn
10 L/min_Left	5.08	13.96	20.30	3.92	14.48	11.70	9.51	17.11	13.37	16.49	14.84	15.39
10 L/min_Right	5.00	18.76	11.26	14.77	14.77	11.07	4.56	16.74	15.61	12.43	12.07	10.87
20 L/min_Left	11.99	14.99	11.99	5.82	11.64	13.31	9.35	25.23	12.27	8.85	15.55	9.65
20 L/min_Right	16.71	10.74	11.46	8.22	13.43	15.99	6.53	14.68	13.70	9.81	17.74	12.08
30 L/min_Left	4.16	14.71	20.41	**2.34**	**20.31**	**18.28**	13.33	18.00	17.33	12.07	15.69	10.98
30 L/min_Right	3.93	20.44	14.15	**2.67**	**20.92**	**19.34**	9.34	18.69	12.15	12.74	15.92	16.56

## Data Availability

The data presented in this study are available upon request from the corresponding author. The data are not publicly available due to laboratory regulations.
